# Temporomandibular Joint Ankylosis as a Sequel of an Overlooked Condylar Fracture in a Child

**DOI:** 10.1155/2024/5101486

**Published:** 2024-01-05

**Authors:** Manel Gharbi, Rym Kammoun, Imen Chaabani, Touhami Ben Alaya

**Affiliations:** ^1^Department of Radiology, University Dental Clinic, University of Monastir, Monastir, Tunisia; ^2^Unit of Bioactive Natural Substances and Biotechnology, Faculty of Dental Medicine of Monastir, University of Monastir, Monastir, Tunisia; ^3^Laboratory of Histology and Embryology, Faculty of Dental Medicine of Monastir, University of Monastir, Monastir, Tunisia; ^4^ABCDF Laboratory for Biological Clinical and Dento-Facial Approach, Faculty of Dental Medicine of Monastir, University of Monastir, Monastir, Tunisia

## Abstract

Temporomandibular joint ankylosis is an important entity that dentists and maxillofacial surgeons should know about. It clinically manifests through a permanent limitation of mandibular movements coupled with mouth opening inferior to 3 cm. This serious pathology can have serious functional repercussions, such as mastication problems, speech troubles, eating disorders, and jaw growth hindrance, in addition to the psychological difficulties in coping with such a condition in daily life. Herein, we present a radiological and chronological illustration of the evolution of temporomandibular joint ankylosis following an overlooked traumatic fracture of the mandibular condyle. The present case report involves an 8-year-old patient referred for a gradually evolving mouth opening limitation following a car accident. Tomodensitometry was helpful as it revealed an osseous block between the left temporomandibular joint surfaces, showing an ankylosis. Posttraumatic cerebral computed tomography scan was performed. It revealed an undetected fracture of the left condyle. The aim of this paper was to show how a traumatic ankylosis could have been avoided if enough attention was paid to the interpretation of immediate posttraumatic computed tomography scans. A thorough dental examination must be carried out once vital emergency is over. Early diagnosis of temporomandibular joint trauma is a crucial factor in preventing complications, such as ankylosis and its consequent oral dysfunctions. The dentist must automatically suspect condylar fracture when a child presents a history of head trauma, especially a mandibular trauma. This case should be a reminder that although temporomandibular joints are very often left out in patients' vital emergency first examination, temporomandibular joints/they are still a highly important structure which omission, and thus, dysfunction, if lesions are present, can lead to nonnegligible medico-legal consequences/that temporomandibular joints should be taken into account during patients' vital emergency first examination because if they are neglected, in the presence of lesions, they cause dysfunction, thus leading to nonnegligible medico-legal consequences.

## 1. Introduction

Mandibular fractures are the most commonly encountered type of maxillofacial fractures in children. Condylar fractures account for 39% to 52% of all mandibular fractures according to some papers. They even reach 70% according to some authors [1].

A large head, a thin cortical bone, and a fragile narrow neck are all features of condyle anatomy in children. They increase risks of fracture especially in mandibular trauma contexts [2].

Condylar fractures are usually caused by an indirect blow to the chin or the mandibular angle. In the absence of pain or the inability to express suffering, lesions can be overlooked and not diagnosed until a complication appears [1, 3]. Delayed and inadequate treatment can have serious repercussions, including malocclusion, jaw growth disorders, and facial asymmetry. In some cases, ankylosis can be noted [4].

Herein, we present the role of imaging modalities in the diagnosis, early detection of possible complications of traumatic fractures involving temporomandibular joints (TMJ), and patients' follow-up.

This paper also highlights the importance of careful interpretation of radiological examinations in preventing condylar fracture complications, especially in pediatric patients.

## 2. Case Presentation

An 8-year-old boy was referred to the oral radiology department for further exploration of severe mouth opening limitation (10 mm), evolving gradually over the previous 3 years with no history of pain episodes reported by the patient or his parents. The patient's caregivers mentioned that he had a car accident at the age of 5 and that he started to get skinner and skinner since then.

**Figure 1:**
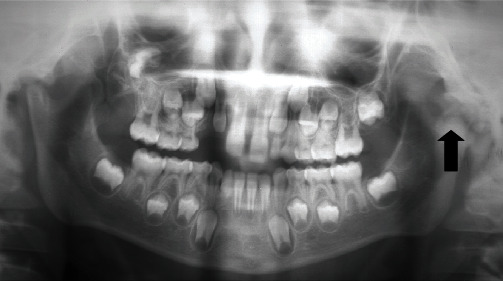
Panoramic radiograph showing ankylotic mass on the left condyle blurring the joint space (black arrow).

**Figure 2:**
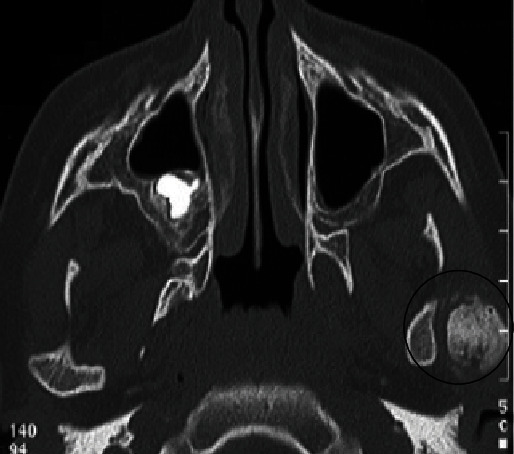
Axial computed tomography slice in bone rendering showing ankylotic mass (black circle) located laterally to the left condyle.

**Figure 3:**
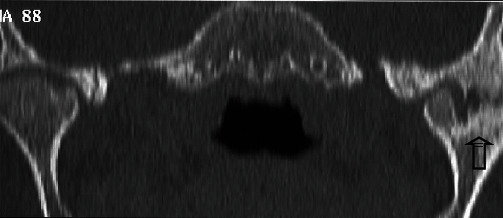
Frontal computed tomography reconstruction in bone rendering showing alteration in articular surfaces of the lateral aspect of the left temporomandibular joint. Note the persistence of joint space in the medial aspect (hatched black arrow).

Radiological explorations were performed. Panoramic radiography did not allow a good visualization of the left TMJ as this area was blurred (Figure 1). Computed tomography (CT) scan of TMJs was helpful in showing details of the TMJ osseous structures (Figures 2 and 3). Scans revealed some morphological deformities in the temporal and condylar articular surfaces and irregularities in the joint space in the left TMJ. These findings were in favor of an ankylosis of the left TMJ, probably occurring after the trauma caused by the car accident.

**Figure 4:**
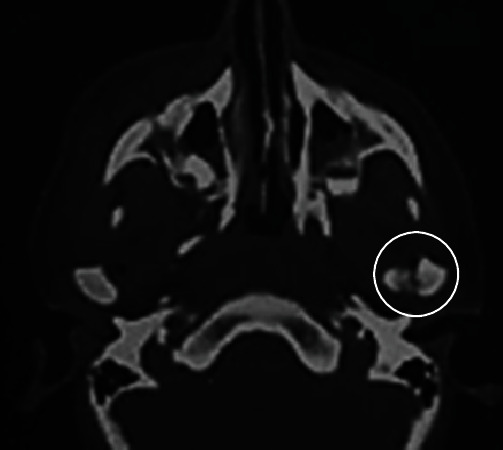
Immediate posttraumatic computed tomography scan. Axial slice in bone rendering showing fracture of the medial pole of the left condyle (white circle).

Anterior cerebral CT scan, performed on the day of the accident, revealed a fracture of the left condyle that was overlooked as the patient reported no pain in that area at that time (Figure 4).

After obtaining a written permission from the patient's parents to use medical records for scientific publication and consent to treatment plan, a surgery involving the left TMJ was carried out under general anesthesia. It consisted in eliminating the bony mass and smoothing both the temporal and condylar articular surfaces, thus recreating the lost joint space which immediately allowed a degree of freedom for the condyle within the articulation.

Anti-inflammatory medication and muscle relaxers were prescribed and taken over 1 month to avoid postsurgical trismus.

Physiotherapy was indicated right after the surgery in order to regain normal mouth opening. The patient was asked to perform tasks several times a day, including movements of protrusion, retrusion, lateral deviation, and mouth opening using tongue depressors.

After 3 months of rehabilitation, the patient regained a maximum interincisal opening reaching 30 mm. He was able to communicate via speech and to eat solid food without any discomfort. His weight ameliorated, and his school results improved remarkably.

The long-term follow-up could not be carried out as the patient did not show up for check-up sessions.

## 3. Discussion

In this paper, a chronological CT illustration of TMJ ankylosis which is not commonly found in the literature is presented. The pictures emphasize the importance of radiological archive as a fundamental pillar of medico-legal justification in cases of complications.

Ankylosis is usually painless. Clinical findings do not reveal any joint sound. Ankylosis can develop secondary to trauma as it causes extravasation of blood into the involved joint, leading to disruption of the fibrocartilage integrity and therefore to an increase in fibrous connective tissue [1, 3, 5]. In the present case, the etiology of ankylosis was related to a previous head trauma in which condylar fracture was overlooked.

The ankylotic mass can be mistaken for a benign fibroosseous tumor (osteochondroma or osteoma) [6, 7]. In the present case, the diagnosis of ankylosis was evident given the clinical and radiological context.

It is important to know that these tumors do not invade the joint space which remains visible. The absence of trauma history or other joint diseases (infectious or autoimmune conditions) can help to differentiate this condition from others.

Fibrous ankylosis can also resemble bony ankylosis because of the presence of hypomobility. However, it is the presence or absence of the articular space that differentiates them.

Several classifications were proposed in order to properly assess the extension of ankylosis and therefore to establish an adequate treatment plan, including the surgical method and the nature and quantity of the TMJ reconstruction materials [8–10].

The most known classification of TMJ ankylosis was established by Sawhney, proposing 4 types of pathological alterations of the joint elements. Although it gives an objective insight into the bony surface remodeling, this classification is still insufficient with regard to a precise evaluation of the evolution of ankylosis, which may strongly impact the treatment outcomes.

It is worth noting that other recognized classifications of TMJ ankylosis are available. The categories of El-Hakim et al. are based on CT scans, dealing with the morphological changes of TMJ anatomical elements as well as the proximity of the ankylosed mass to the adjacent vital structures, especially the maxillary artery, thus allowing the surgeon to elaborate an adequate surgical treatment plan and to achieve better operative results with the fewest complications possible [8]. Another classification aiming to assess the medial displacement of condyles was proposed by He et al. [10].

For our patient, ankylosis was fibrous as a thin joint space was still visible on CT scans. It was classified as Type II according to both classifications.

In general, panoramic radiographs reveal TMJ deformity and complete absence of the joint space obliterated with a bone formation bridging the ramus and the zygomatic arch [1].

The projection of the superior airways frequently crosses over the condylar neck, producing a thin radiolucent line that may be misleading, especially in the context of trauma.

In the present case, on the day of the accident, the patient had a nonreadable panoramic due to superimpositions. The panoramic performed 3 years later to further explore the patient's mouth limitation was confronted with the clinical findings, and the diagnosis of ankylosis was then made.

With high resolution and using multiplanar reconstructions, the CT scan provides further data about the anatomical elements and the surrounding environment of TMJ. It also provides details about the morphological changes as it assesses both the medial and lateral poles as well as the region in-between without overlap [1, 11].

In cases of fibrous ankylosis, CT and CBCT (cone beam computed tomography) usually reveal a limited or absent condylar translation as the joint space is narrowed. Cortical bone may show irregularities. With regard to cases of osseous ankylosis, CT findings include partial or complete obliteration of the joint space by a small or a large osseous mass that may fuse the condyle and the temporal fossa [12].

For our patient, CT was performed because a cerebral lesion was suspected. This tool allowed the visualization of the fractured condyle which was previously overlooked.

CBCT is less expensive and irradiating than CT, especially for pediatric patients, but it is not used for explorations in emergency cases because children are generally not cooperative, leading to movement artifacts [13–15].

In the present case, the condylar fracture took place in the context of a head trauma. Priority was therefore given to the exploration of possible cerebral lesions, and CT was the tool of choice. Besides, CBCT was not performed as it was not available in our hospitals in the 90s.

To the best of the authors' knowledge, only few publications have presented a radiological illustration of the evolution of condylar fractures to ankylosis.

The distance between the ankylotic mass and some important structures, such as the internal maxillary artery, mandibular foramen, lateral pterygoid plate, and external auditory canal, should be considered before any intervention. CT studies have also revealed the involvement of the glenoid fossa, foramen ovale, jugular foramen, and mastoid bone in the ankylotic mass [1, 16, 17].

TMJ ankylosis in children can be a deterrent to normal mandibular growth, especially in the presence of a bilateral problem giving the young patient a “Bird face” appearance. The sequelae become more visible as the child grows [16, 18, 19].

The complications related to TMJ ankylosis include several oral dysfunctions. Moreover, this pathology deeply affects the child's facial skeletal development. Facial dysmorphosis caused by an early traumatic ankylosis can cause psychological stress and therefore negatively impacts the patient's quality of life [5, 20]. Surgical treatment proves to offer an overall improvement in the pediatric patients' welfare, which is confirmed by their caregivers [21, 22].

This therapeutic choice entails risks of injury to the facial nerve, middle meningeal artery, and maxillary artery [17, 23, 24].

In this case, the patient's maximal mouth opening was severely affected due to ankylosis as it was restricted to 10 mm. This hugely hindered the patient's ability to make mandibular movements and thus to adequately eat various foods (he was only consuming liquid food), resulting in a progressive weight loss of the child. He was incapable of properly communicating with peers at school. As the patient was an infant, mandibular growth was slowed down, leading to both gnathic and dental malocclusion. Indeed, the patient was suffering from a mandibular retrognathism and a unilateral cross-bite of the left posterior teeth. Other complications may include obstructive sleep apnea if ankylosis persists in the long term [25].

Multidimensional radiological examination of traumatized patients should focus on the search for condylar and subcondylar fractures to avoid risks of ankylosis.

CT allows a thorough study of the fracture lines and their orientation. Matching the reconstruction plane and the fracture line helps to visualize the fracture in all its length. Narrow window allows visualization of TMJ fracture inflammatory and/or infectious complications in the surrounding soft tissues (thickening/abscess of the lateral pterygoid, medial pterygoid, masseter, and temporal or septation of subcutaneous fat).

Once TMJ fracture is diagnosed, and particularly in cases of pediatric patients, a multidisciplinary decision to treat should be immediately taken and discussed with the patient's family [26], thus avoiding any possible alterations in the child's facial and overall growth [5, 18, 19, 27, 28].

In the present case, if a jaw mobilizer was indicated right after the trauma, ankylosis would have been minimized, if not avoided. Indeed, in the cerebral CT performed right after the trauma, a fracture in the head of the condyle was visible, but it was overlooked.

## 4. Conclusion

TMJ ankylosis can have a negative impact on facial growth in young patients and can therefore be a huge impairment to a person's physical and psychological development as many functions can be affected by this disorder, mainly mastication, speech, and swallowing [29].

The aim of this paper was to show the crucial need for suspecting and establishing the diagnosis of condylar fractures within an adequate timeframe, especially for children having a trauma history, even in the absence of external signs of head injury. This implies the medical responsibility of radiologists, dentists, and other healthcare practitioners involved.

Any pediatric patient presenting to the dental office or to the emergencies with a head trauma context involving mainly the mandible must be oriented for further exploration of TMJ by CT or CBCT scans. The latter are becoming more available and accurate and are considered more economic, irradiation-wise, and financially-wise.

It is important to note that early physiotherapy should be immediately indicated to restore normal masticatory activity, continue mandibular growth, and thus prevent ankylosis [30].

We recommend that traumatized patients engage in physiotherapy as a prophylactic measure even if TMJ radiological examinations are not conclusive.

A long follow-up period extending until the end of mandibular growth must be associated with surgical treatment in order to detect early signs of recurrence which remains possible due to the high regenerative and remodeling capacity in children [29, 31].
